# Promoter-enhancer looping and shadow enhancers of the mouse αA-crystallin locus

**DOI:** 10.1242/bio.036897

**Published:** 2018-11-07

**Authors:** Rebecca S. McGreal-Estrada, Louise V. Wolf, Ales Cvekl

**Affiliations:** 1 Departments Ophthalmology and Visual Sciences and Department of Genetics, Albert Einstein College of Medicine, 1300 Morris Park Ave, Ullmann 123, Bronx, NY 10461, USA; 2 Office of Research Services (ORS), Icahn School of Medicine at Mount Sinai, One Gustave L. Levy Place – Box 1120, New York, NY 10029-6574

**Keywords:** Chromatin looping, αA-crystallin, Enhancer, Shadow enhancer, Lens, Development

## Abstract

Gene regulation by enhancers is important for precise temporal and spatial gene expression. Enhancers can drive gene expression regardless of their location, orientation or distance from the promoter. Changes in chromatin conformation and chromatin looping occur to bring the promoter and enhancers into close proximity. αA-crystallin ranks among one of the most abundantly expressed genes and proteins in the mammalian lens. The αA-crystallin locus is characterized by a 16 kb chromatin domain marked by two distal enhancers, 5′ DCR1 and 3′ DCR3. Here we used chromatin conformation capture (3C) analysis and transgenic approaches to analyze temporal control of the mouse αA-crystallin gene. We find that DCR1 is necessary, but not sufficient alone to drive expression at E10.5 in the mouse lens pit. Chromatin looping revealed interaction between the promoter and the region 3′ to DCR1, identifying a novel enhancer region in the αA-crystallin locus. We determined that this novel enhancer region, DCR1S, recapitulates the temporal control by DCR1. Acting as shadow enhancers, DCR1 and DCR1S are able to control expression in the lens vesicle at E11.5. It remains to be elucidated however, which region of the αA-crystallin locus is responsible for expression in the lens pit at E10.5.

## Abstract (precis)

**Summary:** The αA-crystallin ranks amongst the most highly expressed tissue-specific genes. It is an advantageous model system to probe both promoter-enhancer looping and to identify distal enhancers and their temporal/spatial activities.

## INTRODUCTION

Precise regulation of gene transcription during tissue development lays the foundation for cellular identity, with levels of gene expression varying greatly between cell types as well as developmental time points (Regev et al., 2017). Important regulatory elements for tissue- and developmental-specific transcription are the enhancers: *cis*-regulatory elements comprised of clustered arrays of transcription factor binding sites (see Barolo and Posakony, 2002; Catarino and Stark, 2018; Long et al., 2016). These ‘classical’ enhancers can drive transcription regardless of their location, orientation or distance from the gene promoter (reviewed in Schaffner, 2015). In recent years, a number of transcriptional units have been found to originate from within many enhancers, and their corresponding transcripts are called eRNAs (Lam et al., 2014). Although prediction of enhancer regions is becoming a routine process by way of genome-wide chromatin profiling methods to identify ‘open’ chromatin regions as well as enhancer associated histone marks (Andrey and Mundlos, 2017), elucidation of the precise cell-specificity and temporal/spatial activities of individual candidate enhancers requires experimentation.

Recent studies of genome organization and evolution coupled with systemic analysis of predicted enhancers in model loci revealed apparent redundancy amongst two or more ‘shadow enhancers’ (Lagha et al., 2012). This term was coined by Levine and co-workers following discovery of multiple enhancers with similar activities (Hong et al., 2008). Shadow enhancers seem to be pervasive, at least in the *Drosophila* genome (Cannavò et al., 2016) and are excellent sources of evolutionary novelty (Hong et al., 2008). In mammalian systems, shadow enhancers were established in *Hox* genes (Nolte et al., 2013) and *Pax6* locus (Antosova et al., 2016). Enhancers involved in limb development have also been shown to act as shadow enhancers and it has been suggested that they are imperative for phenotypic robustness (Osterwalder et al., 2018). For example, shadow enhancers under normal conditions may exhibit redundancy, each being able to induce similar gene expression, however under stress conditions, both enhancers may be required to maintain vigorous regulation (Frankel et al., 2010; Perry et al., 2010). Thus, the area of transcriptional control is poised for novel discoveries underlying complexity of gene control *in vivo*, including enhancer syntax and regulatory genome evolution (Farley et al., 2016).

A hallmark feature of enhancers is physical tethering between the promoter and distal enhancer(s) and formation of DNA loops (Long et al., 2016) that are further integrated into a 3D organization of chromatin that is thought to be important for tissue specific gene regulation (Rao et al., 2014). It has been shown that chromatin is organized into defined functional units to mediate the effects of *cis*-regulatory elements by both long and short-range interactions (Rao et al., 2014). Although chromatin looping has been shown to be important for tissue specific gene regulation, little work has been performed to elucidate the changes that occur in chromatin structure and subsequent effects on gene regulation and expression during cellular differentiation.

An advantageous tissue to study transcription, chromatin dynamics and subnuclear organization and compartmentalization during mammalian development is the ocular lens. The mature lens consists of an anterior layer of epithelial cells that overlies the bulk of the lens, made up of differentiated fiber cells. This compartmentalization of the lens into epithelium and fibers initiates from an early transitional structure termed the lens vesicle (Lovicu et al., 2011). Differentiating cells elongate towards the anterior of the vesicle and fill the void to become the primary fiber cells. The cells at the anterior of the lens vesicle differentiate into a sheet of single-layered cuboidal epithelial cells, and those close to the lens equator will divide continually, eventually exiting cell cycle and becoming secondary lens fiber cells. The fiber cells in the center of the lens are required to lose their organelles, including the nuclei, in order to prevent light scattering and maintain the transparency of the lens (see Bassnett, 2009).

Crystallins are the most abundant proteins in terminally differentiated lens fiber cells (Bassnett et al., 2011). Their high level of expression, along with their distinct spatial distribution is essential for the transparency and refractive function of the lens. Together with globin genes in erythrocytes and calcium channel subunit Cacna2d in neurons, the crystallins rank amongst the most highly expressed genes in mammalian tissues (Sun et al., 2015a) and serve as an advantageous model to study fundamental principles of transcription during cellular differentiation (Limi et al., 2018). The most abundant αA-crystallin is a small heat shock chaperone protein and represents up to 17% of all newborn mouse lens water-soluble proteins. The αA-crystallin (*Cryaa*) gene evolved from gene duplication of the αB-crystallin (*Cryab*) that evolved from Hspb1-like ancestral gene (Cvekl et al., 2017). Loss of αA-crystallin leads to lens opacification and cataract (Andley, 2007; Bloemendal et al., 2004; Rivier et al., 1999). In lens, αA-crystallin gene expression initiates in the invaginating lens placode followed by uniform expression in the lens vesicle and subsequent primary lens fiber cell differentiation is associated with dramatic upregulation of αA-crystallin expression (Robinson and Overbeek, 1996). Thus, studies of transcriptional regulation of αA-crystallin gene are critical to understand lens morphogenesis.

**Fig. 1.**
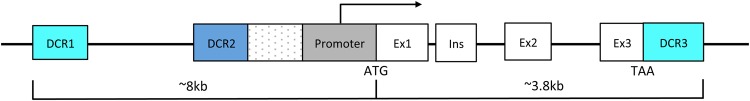
**Schematic representation of the mouse αA-crystallin locus.** Genomic organization of the αA-crystallin gene identifying the locations of the evolutionarily conserved DCR1 (−7706 to −7492), DCR2 (−1900 to −1670) and DCR3 (+3650 to +3656). Three exons (Ex1, Ex2 and Ex3) as well as a rodent specific exon (Ins) are also shown.

The current model of αA-crystallin transcriptional control includes lens-fiber cell specific promoter fragment (−366/+46) (Overbeek et al., 1985) and a pair of evolutionarily conserved distal enhancers, 5′-DCR1 and 3′-DCR3 (Fig. 1). It was found that the −8 kb DCR1 region directs early expression beginning in the lens vesicle at E11.5 and DCR3's weaker activity is delayed by 24 h (Yang et al., 2006). The 1.9 kb promoter, including DCR2 at its 5′-end, supports a weak expression at even later stages of lens formation and is highly prone to positional effects while the presence of both DCR1 and DCR3 nearly eliminates this effect (Yang et al., 2006). However, endogenous αA-crystallin expression is first apparent at E10.5 (Robinson and Overbeek, 1996). To resolve insufficiency of DCR1/3, transgenic mice were generated using BAC constructs and a 15 kb αA-crystallin locus lacking DCR3 (Wolf et al., 2008). It was found that a 15 kb αA-crystallin fragment is sufficient for the earliest expression in the lens pit at E10.5. In this study, we use chromatin conformation analysis as well as transgenic mouse models to dissect further the transcriptional regulation of the αA-crystallin locus. Our central goal was to probe promoter-enhancer looping in lens and non-lens cells and take this conformational information to predict additional candidate enhancers.

## RESULTS

### DCR1 is required, but not sufficient, for early expression of αA-crystallin

**Fig. 2.**
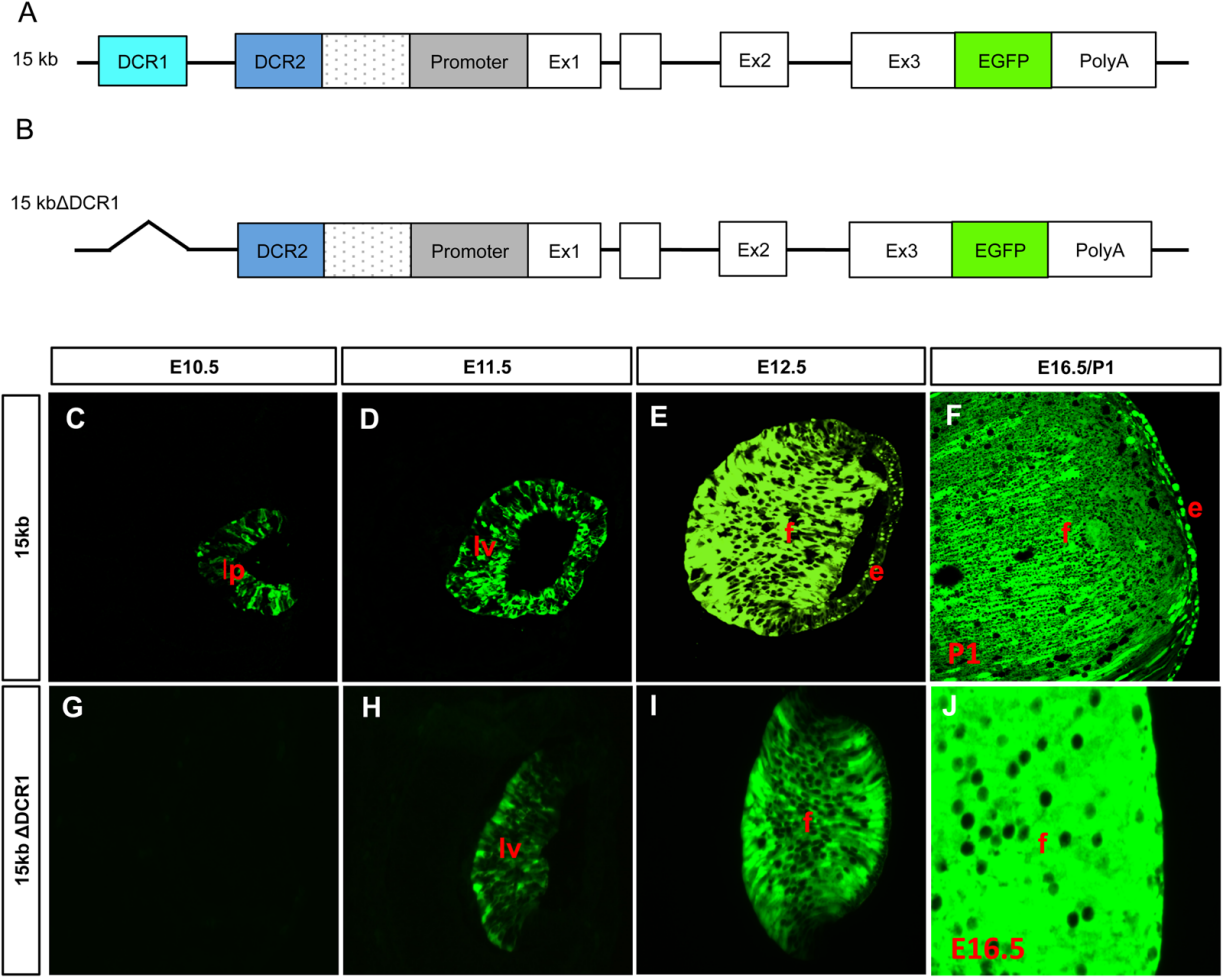
**15 kb and 15 kb ΔDCR1 transgene expression in the mouse lens.** (A–B) Diagrammatic representation of a 14 kb region αA-crystallin locus with an EGFP/*polyA* insert to generate a 15 kb construct (A) and with DCR1 deleted (B). (C–J) Expression of EGFP from the 15 kb construct is first seen at E10.5 in the lens pit (C) and in the lens vesicle at E11.5 (D). Expression is upregulated in E12.5 lens fiber cells and expression is also apparent in the lens epithelium (E). Strong expression continues in the fiber cells at newborn stage P1 (F). Expression of EGFP from the 15 kb ΔDCR1 construct is absent in the lens pit at E10.5 (G) with expression initiating in the lens vesicle at E11.5 (H). Increased expression continues in the fiber cells at E14.5 but is not observed in the epithelium (I). Expression continues further in E16.5 fiber cells (J). Lens epithelium, e; lens fiber cells, f; lens pit, lp; lens vesicle, lv.

Previous studies of the temporal regulation of αA-crystallin did not identify the DNA region necessary for its earliest expression in the lens pit (Yang et al., 2006). Exogenous expression of EGFP driven by a 15 kb αA-crystallin locus (Fig. 2A), allowed for recapitulation of the endogenous αA-crystallin expression (Wolf et al., 2008), with expression beginning in the lens pit at E10.5 (Fig. 2C–F). It remains to be elucidated, however, the exact region of the αA-crystallin locus responsible for earliest expression of αA-crystallin at this stage. The DCR1 regulatory region of the αA-crystallin locus alone has previously been demonstrated to be insufficient to drive expression at E10.5 in transgenic mice (Yang et al., 2006), we therefore generated a mouse line that used EGFP driven by the 15 kb genomic region of the αA-crystallin locus with the DCR1 region deleted (Fig. 2B). Unexpectedly, even when the entire locus is present, the absence of DCR1 delayed the expression of EGFP (Fig. 2G–J), with no EGFP expression being seen at E10.5 in the lens pit (Fig. 2G). These results suggest that both DCR1 as well as another region of this 15 kb genomic region is required for expression of αA-crystallin in the lens pit.

### Identification of an additional control region (DCR1S) in the mouse αA-crystallin locus

**Fig. 3.**
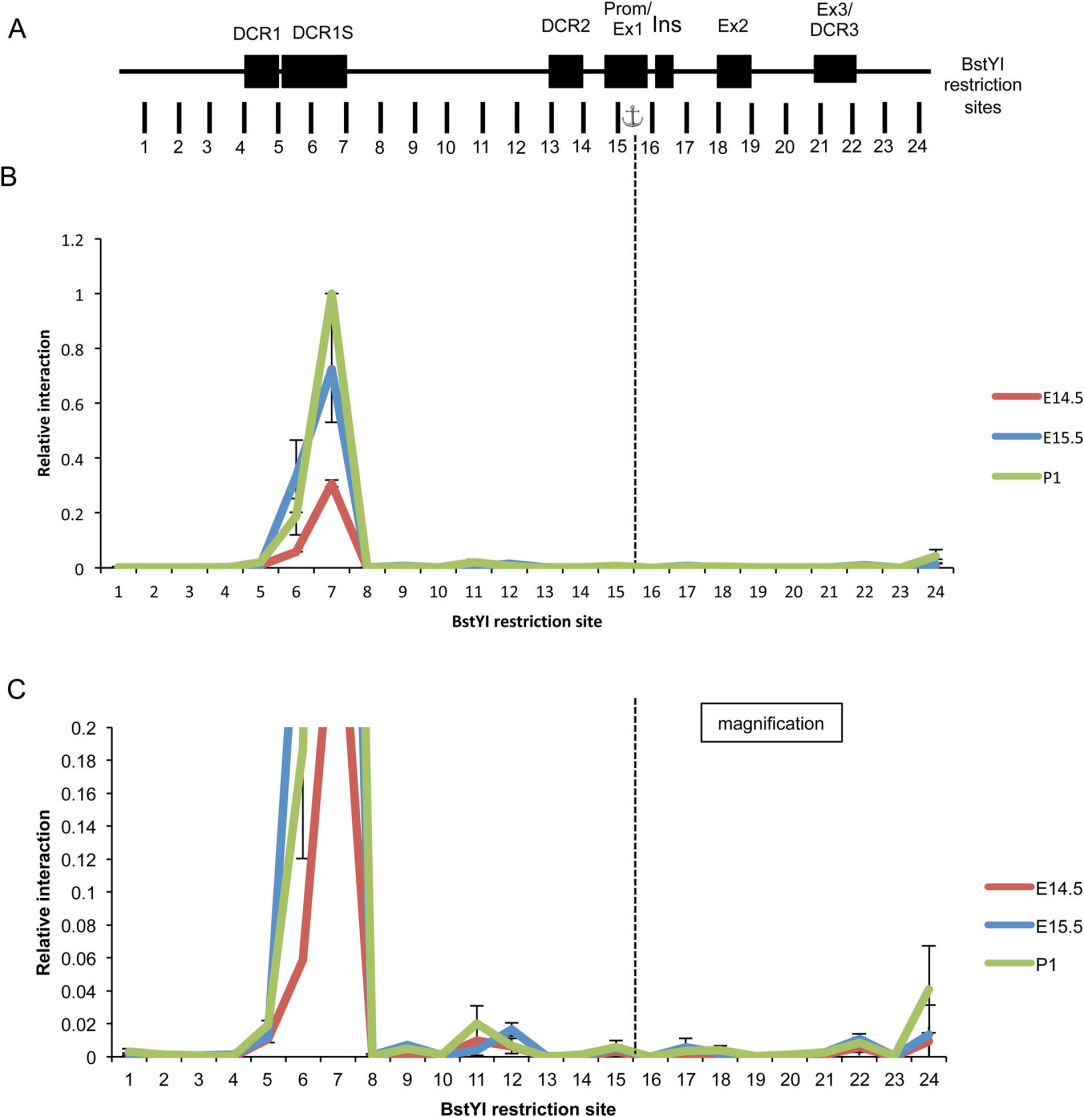
**3C mapping of chromatin interactions in the αA-crystallin locus of E14.5, E15.5 and P1 mouse lens.** (A) Genomic organization of the αA-crystallin locus, spanning 16 kb. Dark boxes represent locations of the evolutionarily conserved DCR1, DCR1S, DCR2, the promoter with adjacent exon 1, the rodent specific exon (Ins), exon 2 and exon 3 with adjacent DCR3. Vertical markers represent locations and numbers of BstYI restriction sites analyzed in this study, with the fragments assayed for variable interaction with the promoter fragment (anchor). Graphical representation is not to scale. (B) Relative cross-linking frequency of regions interacting with the αA-crystallin promoter with relative interaction plotted on the y-axis and restriction digest fragment number on the x-axis. (C) Magnification of panel (B) with reduced y-axis scale to identify less frequent interactions. Dashed line shows position of anchor fragment. Each value is derived from three biological samples (*n*=3) and the standard errors are indicated. Values are normalized to P1=1.0.

Recent work has demonstrated the importance of chromatin conformation on gene expression with significant progress being made in determining looping that occurs within gene loci to allow for interactions between enhancer and promoter regions required for gene transcription (Frost et al., 2018; Jiang and Peterlin, 2008; Miele and Dekker, 2008; Vakoc et al., 2005). To determine other regions of the αA-crystallin locus important for gene expression we used the chromatin conformation capture (3C) assay (Dekker et al., 2002) to examine looping that occurs at the αA-crystallin locus during lens development. Interactions between the αA-crystallin promoter (anchor, Fig. 3A) and the rest of the locus were measured and relative interaction frequency determined using chromatin derived from mouse lenses at three stages, including E14.5, E15.5 and P1 (*n*=3). It was observed that a significant interaction occurs between the promoter and a large portion of the chromatin 3′ adjacent to DCR1 (Fig. 3B). This interaction was significantly lower in the earlier stage observed at E14.5. There were also smaller interactions observed 5′ adjacent to the DCR2 region (Fig. 3C) as well as at the DCR3 region and the distal 3′ end of the locus which was much more prominent in the P1 lens. We also analyzed chromatin from mouse lenses at stages E16.5 and E17.5 (*n*=2) and found a similar broadened peak at the DCR1 region as well as the 3′ distal peak (Fig. S1). We extended the region examined by 10 kb 3′ beyond this distal peak and observed no further interaction peaks (data not shown). Based on this data we hypothesized that the region 3′ to DCR1 (−7492 to −6039 from transcription start site; called here DCR1S) could be important for regulation of αA-crystallin expression, possibly at the earlier stages of E10.5.

**Fig. 4.**
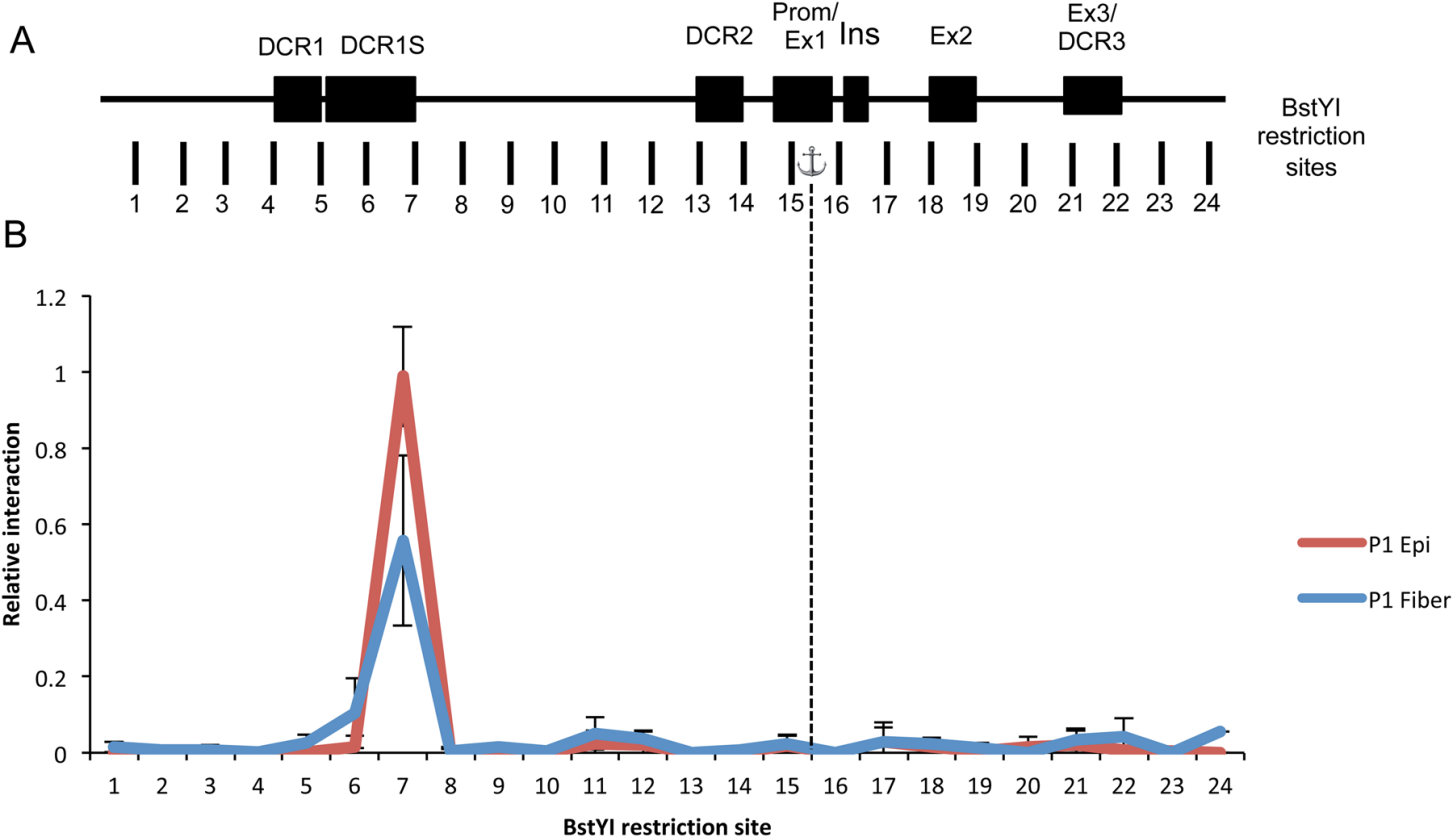
**3C mapping of chromatin interactions in the αA-crystallin locus of micro dissected mouse lens epithelial and fiber tissue.** (A) Genomic organization of the αA-crystallin locus, spanning 16 kb. Dark boxes represent locations of the evolutionarily conserved DCR1, DCR1S, DCR2, the promoter with adjacent exon 1, the rodent specific exon (Ins), exon 2 and exon 3 with adjacent DCR3. Vertical markers represent locations and numbers of BstYI restriction sites analyzed in this study, with the fragments assayed for variable interaction with the promoter fragment (anchor). Graphical representation is not to scale. (B) Relative cross-linking frequency of regions interacting with the αA-crystallin promoter with relative interaction plotted on the y-axis and restriction digest fragment number on the x-axis. Dashed line shows position of anchor fragment. Each value is derived from three biological samples (*n*=3) and the standard errors are indicated. Values are normalized to P1=1.0.

**Fig. 5.**
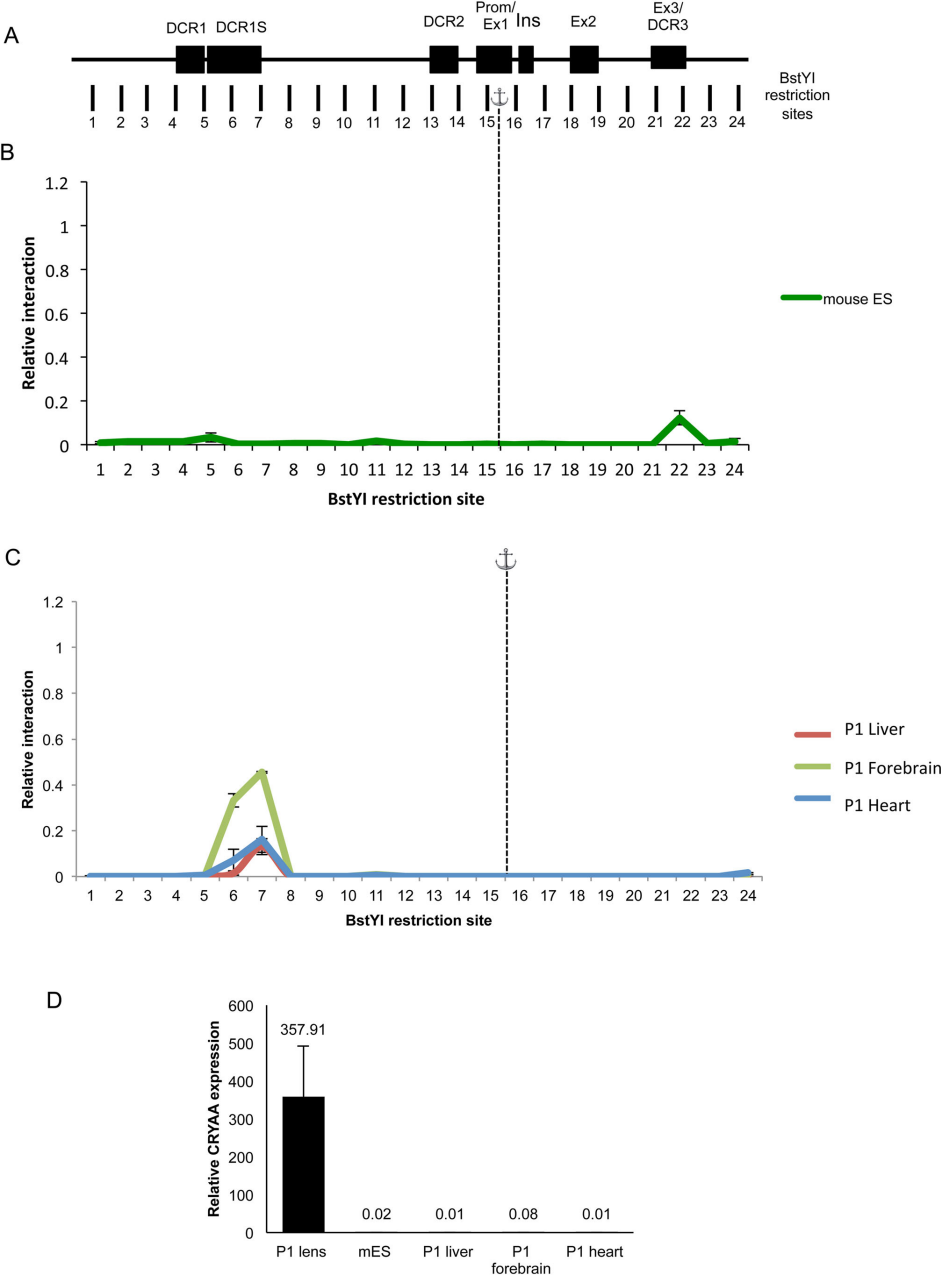
**3C mapping of chromatin interactions in the αA-crystallin locus of mouse embryonic stem cells and other non-αA-crystallin expressing tissues.** (A) Genomic organization of the αA-crystallin locus, spanning 16 kb. Dark boxes represent locations of the evolutionarily conserved DCR1, DCR1S, DCR2, the promoter with adjacent exon 1, the rodent specific exon (Ins), exon 2 and exon 3 with adjacent DCR3. Vertical markers represent locations and numbers of BstYI restriction sites analyzed in this study, with the fragments assayed for variable interaction with the promoter fragment (anchor). Graphical representation is not to scale. (B–C) Relative cross-linking frequency of regions interacting with the αA-crystallin promoter of (B) mouse embryonic stem cells and (C) P1 mouse liver, forebrain and heart tissue. Relative interaction plotted on the y-axis and restriction digest fragment number on the x-axis. Dashed line shows position of anchor fragment. Each value is derived from three biological samples (*n*=3) and the standard errors are indicated. Values are normalized to P1=1.0. (D) Bar chart exhibiting relative levels of αA-crystallin expression in P1 mouse tissues and mouse embryonic stem cells (*n*=3; error bars=±standard deviation).

To determine if there are any differences between two lens cell types, we next examined looping in chromatin from microdissected newborn (P1) lens epithelium and fiber cells (Fig. 4). As with other crystallins, the expression of αA-crystallin is much higher in lens fiber cells compared to lens epithelium (Sun et al., 2015a; Zhao et al., 2018a), therefore we hypothesized that we would see differences in the looping pattern of the chromatin. We found that the interaction profile of the αA-crystallin locus was not significantly different between lens epithelium and lens fibers (Fig. 4B), despite the differences in expression levels. To extend these studies, we performed the 3C assay on mouse embryonic stem (ES) cell line to determine whether this looping was present when the cells were in a completely undifferentiated state. Very limited DNA looping was observed at the αA-crystallin locus in chromatin isolated from ES cells (Fig. 5B). Finally, we performed the 3C assay on chromatin from non-lens tissues, including heart, liver and forebrain of P1 mice, where αA-crystallin expression is absent (Fig. 5D). Interestingly, albeit at a much lower frequency, the major interaction peak that occurs in lens around the DCR1 region also exists in these differentiated non-lens tissues (Fig. 5C).

### DCR1 and DCR1S act as shadow enhancers, conferring similar temporal and spatial expression in lens vesicle, lens epithelium and primary lens fibers

**Fig. 6.**
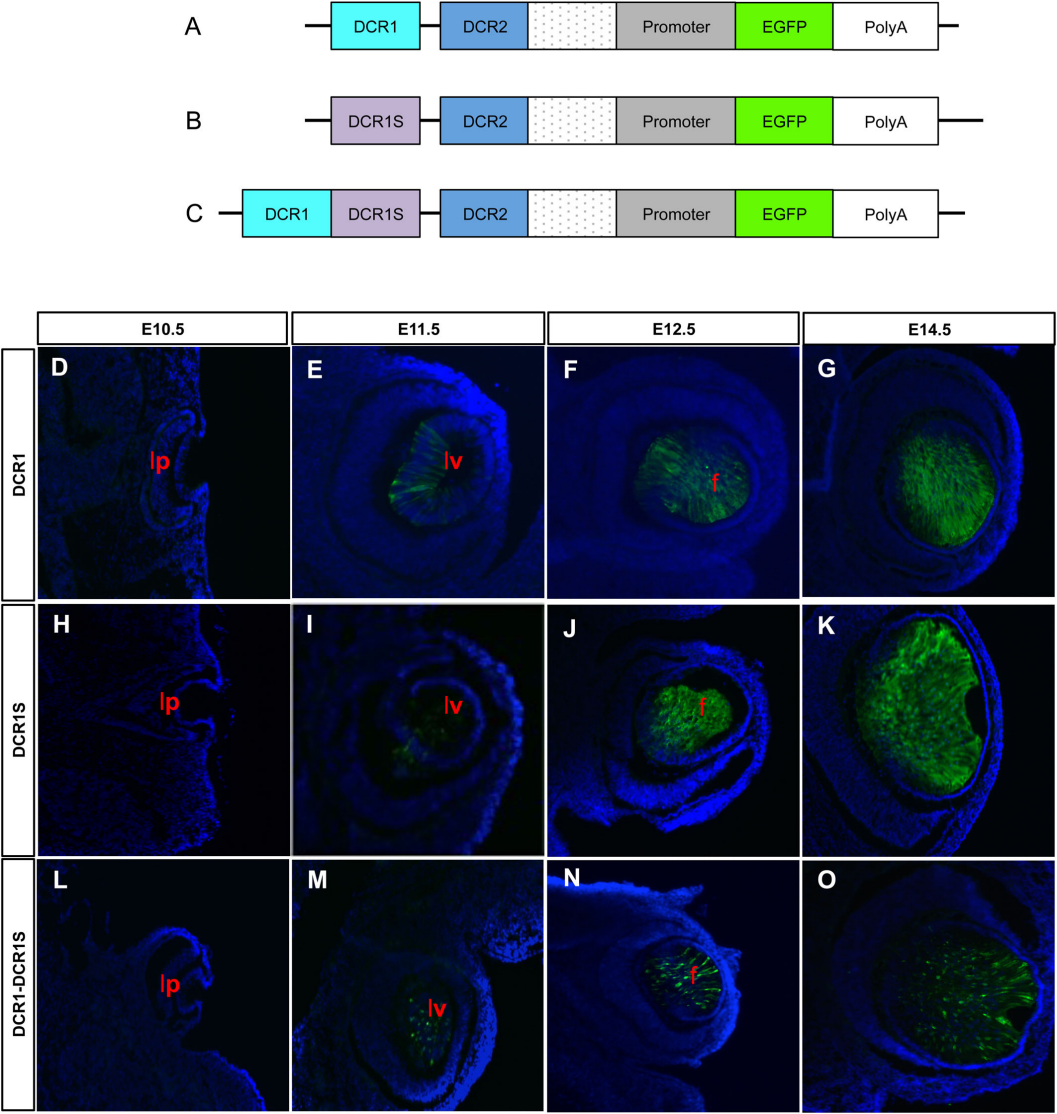
**Expression of EGFP driven by three transgenic constructs in the developing mouse eye.** (A–C) Schematic diagram of constructs used to analyze the function of DCR1 and/or DCR1S *in vivo*. Each contained the 1.9 kb DCR2-promoter fragment and an EGFP reporter as well as (A) DCR1, (B) DCR1S and (C) DCR1 and DCR1S. For all three transgenic lines, EGFP was absent in the lens pit at E10.5 (D,H,L). Expression was first observed at E12.5 for all three lines (E,I,M) and increased in lens fiber cells at E14.5 (F,J,N) and P1 (G,K,O). EGFP could not be seen in the lens epithelium. Lens epithelium, e; lens fiber cells, f; lens pit, lp; lens vesicle, lv.

Prior analysis of mice expressing EGFP under control of the αA-crystallin 1.9 kb genomic region containing the promoter and its 5′-adjacent DCR2 show modest expression in lens fiber cells from E13.5 (Yang et al., 2006). To determine the role of DCR1S in lens development we generated transgenic mice lines expressing EGFP under the control of DCR1 and/or DCR1S in combination with the 1.9 kb extended promoter (Fig. 6). As described previously (Yang et al., 2006), the construct containing DCR1 (Fig. 6A) allows for expression of EGFP beginning at stage E11.5 (Fig. 6D–G). Earlier studies show that in the absence of DCR1, expression is delayed even more severely with onset of EGFP beginning at E13.5 (Yang et al., 2006). Interestingly, in the presence of DCR1S (Fig. 6B), the onset of expression of EGFP is similar to that of DCR1 alone (Fig. 6A), with expression beginning at E11.5 (Fig. 6H–K). We then examined the effect of the presence of both DCR1 and DCR1S (∼2 kb; Fig. 6C) to determine whether these combined regions are sufficient to recapitulate endogenous expression of αA-crystallin in the lens pit. We found however that the converse was true: even in the presence of this 2 kb region comprising DCR1 and DCR1S, expression was still absent in the lens pit (Fig. 6L–O) with expression of EGFP observed from E11.5. These studies were performed with three independent lines obtained for each construct and the results are summarized in Table 1. We conclude that DCR1 and DCR1S function as shadow enhancers required for αA-crystallin gene expression in the lens vesicle (E11.5); however, even together they are insufficient to elicit transgene expression in the lens pit (E10.5).

**Table 1.**
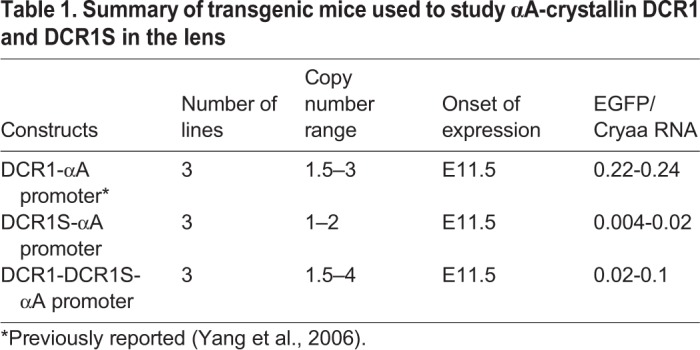
**Summary of transgenic mice used to study αA-crystallin DCR1 and DCR1S in the lens**

## DISCUSSION

In this study we present evidence for DNA looping at the αA-crystallin locus in mouse. Based on this chromatin conformation we subsequently identified a novel enhancer region, which, determined by transgenic mouse studies, is thought to act as a shadow enhancer eliciting expression patterns similar to that of DCR1. Although expression of αA-crystallin is highly tissue-restricted, looping was also found in terminally differentiated non-αA-crystallin expressing tissues, albeit at a lower frequency. In contrast, the pluripotent ES cells do not exhibit any significant physical interactions of distal regions within the αA-crystallin locus.

Due to the short window of time in which it has to produce all proteins required for the life of the organism, lens is an advantageous model tissue in which to study transcriptional control. αA-crystallin in particular, with its extraordinarily high relative expression levels must be highly regulated to generate such massive quantities before fiber cell organelles are degraded (Brennan et al., 2018; Limi et al., 2018). Previous work has identified an αA-crystallin promoter fragment (−364 to +45) that can support expression of a linked CAT gene, but only in fiber cells of the lens beginning after E12.5 (Overbeek et al., 1985), 2 days following endogenous αA-crystallin expression initiates (Robinson and Overbeek, 1996). Three regulatory regions, DCR1, DCR2 and DCR3, have more recently been identified within the αA-crystallin locus using the VISTA algorithm for comparative genomics to determine evolutionarily conserved non-coding regions and studied in transgenic mice (Yang et al., 2006). Using DCR1/1.9 kb promoter/EGFP reporter system, EGFP was detected in the lens vesicle at E11.5, but did not recapitulate endogenous expression at E10.5 in the lens pit. A modified 14 kb region of the αA-crystallin locus (15 kb transgene containing 14 kb of the αA-crystallin genomic locus with a 1 kb insert of EGFP/*polyA*, DCR3 absent; Fig. 2A) however, can support strong EGFP expression in the lens pit of the E10.5 mouse embryo [Fig. 2C; (Wolf et al., 2008)], suggesting that the enhancer region responsible for the earliest expression of αA-crystallin lies within this genomic region. Here, using the previously described 15 kb transgene [Fig. 2A; (Wolf et al., 2008)], with the DCR1 region removed (Fig. 2B), we determined that DCR1 is required for the expression at E10.5, however another portion of this 14 kb genomic region must also be required, as presence of DCR1 alone in the previous studies (Yang et al., 2006) did not allow for expression in the lens pit.

Previous methods to determine possible enhancer regions in the αA-crystallin locus relied upon identification of evolutionarily conserved non-coding regions (Yang et al., 2006). Here we used analysis of chromatin looping to identify novel potential regulatory regions. Our reasoning was that local chromatin looping should point to interacting DNA regions that are less evolutionarily conserved and these studies will be also used to aid in our ongoing 4C/HiC studies of chromatin during lens differentiation. 3C analysis was used here to determine that looping occurs at high levels, indicated by higher interaction frequency peaks, between the αA-crystallin promoter and the region 3′ adjacent to the DCR1 enhancer in lens (Figs 3, 4 and S1), suggesting the existence of a novel 1.5 kb regulatory region, from here on referred to as ‘DCR1S’. Interaction frequency between the promoter and DCR1S was much higher at the later developmental stages of E15.5 and P1 compared to E14.5 (Fig. 3B). This increased interaction frequency could be responsible for the increase in αA-crystallin expression in lens fiber cell compartment by RNA-seq from E14.5 to P0.5 (Zhao et al., 2018b). Interestingly, the interaction frequency between the promoter and DCR1S in fiber cells and epithelium (Fig. 4B) is not significantly different, despite the higher levels of αA-crystallin found in fiber cells compared to epithelium. A known marker of active enhancers is the presence of RNA polymerase II as well as the epigenetic marker H3K27ac. Indeed, we found and reported earlier on these features in the DCR1S region of the αA-crystallin gene (Sun et al., 2015a,b). In addition, previous work from our lab has also shown the particular presence of several transcription factors *in vivo* in the DCR1S region including c-Jun and Etv5 (Xie et al., 2016) as well as ATP-dependent chromatin remodeling enzymes Brg1 and Snf2h (Yang et al., 2006). Coincidentally, conservation is also seen within this 1.5 kb DCR1S region in our previous VISTA alignment studies, albeit not as highly conserved as the DCR1 region. Taken together, these data demonstrate that this DCR1S region physically interacts with the promoter and identify a novel *in vivo* enhancer region of the *Cryaa* locus. Due to difficulty of sample collection, we did not analyze looping at stages prior to E14.4, however we predict a sharp increase in looping at the onset of αA-crystallin expression at E10.5 in the mouse lens.

To further analyze looping at the αA-crystallin locus we performed the 3C assay on mouse ES cells to determine whether looping would occur in cells in which chromatin exists in a ‘ground state’. Looping in embryonic stem cells has been identified between enhancers and core promoters of ES cell-specific genes (Kagey et al., 2010). To be maintained in a pluripotent state, ES cells are required to express their pluripotency factors at high levels whilst repressing lineage specific genes to prevent differentiation. As expected, our data show very little looping occurring at the αA-crystallin locus (Fig. 5B), correlating with the lack of expression in these undifferentiated cells. To determine whether this absence of looping was a feature of αA-crystallin expression or the undifferentiated state of the ES cells, we performed 3C in terminally differentiated tissues (Fig. 5C) that also do not express αA-crystallin (Fig. 5D). Surprisingly however, albeit at very low interaction frequencies, a peak was observed at the DCR1/DCR1S region in all three tissues, including liver, forebrain and heart (Fig. 5C). A possibility exists that transcription across this 2 kb region exists in non-lens cells; however, our attempts to detect any significant ncRNA expression using bioinformatics analyses and cDNAs from these tissues were negative. It is thus possible that in terminally differentiated tissues, less-frequent tethering non-productive interactions may still occur in the absence of ongoing transcription whereby the chromatin is in a ‘poised’ state, but absence of lens associated transcription factors prevents gene expression.

Our initial aim for this project was to find the region of the αA-crystallin locus required for the earliest expression of the gene in the lens pit at E10.5 (Robinson and Overbeek, 1996). To determine whether DCR1S is responsible for this expression we generated transgenic mice with EGFP under the control of DCR1 and/or DCR1S and the 1.9 kb promoter region (Fig. 6A–C). It was found that, although DCR1S could not drive expression of EGFP at E10.5 alone (Fig. 6H) or in combination of DCR1 (Fig. 6L), DCR1S was able to recapitulate expression patterns of DCR1 and drive expression at E11.5 (compare Fig. 6E,I). Alternatively, both DCR1 and DCR1S enhancers are required on their own for the expression of *Cryaa* gene as independent elements. The availability of CRISPR-EZ method (Chen et al., 2016) allows a rigorous analysis to determine both necessity and sufficiency of DCR1, DCR1S and the entire 2 kb region.

The phenomenon of two or more enhancer regions that are able to perform similar functions is known as ‘enhancer redundancy’ (Osterwalder et al., 2018). Studies of other mammalian gene loci have identified enhancers of extremely similar functions and spatiotemporal patterns of activity (Attanasio et al., 2013; Lam et al., 2015; Marinić et al., 2013; Osterwalder et al., 2018). This type of enhancer is also referred to as ‘shadow enhancer’, a term first coined to describe enhancers of similar functions found in *Drosophila* (Cannavò et al., 2016; Frankel et al., 2010; Hong et al., 2008; Perry et al., 2010). The evolutionary purpose of these redundant shadow enhancers still remains to be completely defined. Many enhancers that appear to be redundant for some overlapping functions are actually not complete shadow enhancers. For example, apparent shadow enhancers in the *shavenbaby* (*svb*) (Frankel et al., 2010) and *snail* (Perry et al., 2010) genes appear redundant under normal environmental conditions, however are essential under more stressful conditions. In addition, other enhancers that seem to overlap in activity have been demonstrated to be essential to for precise spatial (Dunipace et al., 2011) or temporal (Dunipace et al., 2013) pattern of expression. Therefore, although DCR1 and DCR1S fulfill minimal criteria to be called shadow enhancers for the temporal expression of αA-crystallin at E11.5, it remains to be elucidated whether they have alternative functions as separate elements.

In addition, in the human αA-crystallin locus, active transcription has been detected at the location corresponding to mouse DCR1 (AP001631.10: hg38 chr21:43,159,066-43,162,227) in non-lens tissues. This suggests the presence of an enhancer RNA (eRNA) transcribed from DCR1. However, our attempts to detect this transcript in mouse tissues based on RNA-seq data and RT-PCR did not reveal the presence of an eRNA (data not shown). Evolutionary differences acting on DCR1 and DCR1S enhancers in mouse and human are likely to explain presence or absence of eRNAs and can be further experimentally tested, including studies of *cis*-regulatory syntax of DCR1 and DCR1S (Farley et al., 2016; Long et al., 2016) and examination of proteins involved in the mediating promoter-enhancer interactions (Cvekl and Pačes, 1992).

In summary, here we identify a novel enhancer region that acts as a shadow enhancer along with DCR1 in the mouse *Cryaa* locus. These enhancers are both capable of inducing expression in the lens vesicle at mouse stage E11.5, however it remains to be elucidated which region of the αA-crystallin locus is responsible for expression in the lens pit at E10.5. Possible candidates include the 3′ distal region for which we see looping with the promoter as well as other evolutionarily conserved noncoding regions identified in introns 1 and 2 (Wolf et al., 2008). Ongoing experiments to map ‘open’ chromatin dynamics during lens differentiation by ATAC-seq are aimed to provide new insights into the unexpected complexity of the *in vivo Cryaa* locus transcriptional control.

## MATERIALS AND METHODS

### Transgenic mice production

Animal husbandry and experiments were conducted in accordance with the approved protocol of the Institutional Animal Care and Use Committee at Albert Einstein College of Medicine and the ARVO Statement for the Use of Animals in Ophthalmic and Vision Research. The αA-BAC constructs were generated as described previously (Wolf et al., 2008). Briefly, the BAC clone RP-23-465G4 was modified using the shuttle vector pLD53.SCAEB to insert EGFP into the third exon of αA-crystallin to generate αA-BAC(ΔDCR3). The 15 kb αA-ΔDCR3-GFP construct (Fig. 2A) was generated by digesting αA-BAC(ΔDCR3) with *XmaI* and *SpeI*. The digests were run on a 0.8% agarose gel overnight and stained with SYBR Gold (Molecular Probes). The 15 kb band was excised from the gel and melted in TAE buffer. The DNA was precipitated in 100% ethanol and sodium acetate (pH 5.2; 300 mM), then washed with 70% ethanol. The 15 kb fragment was cloned into pBluescript SKII vector sites, *XmaI* and *SpeI*. 15 kb αA-ΔDCR1 ΔDCR3-GFP construct (Fig. 2B) was synthesized by GenScript (Piscataway) in a pUC57 vector and subcloned into pBluescript SKII vector sites, *XmaI* and *SpeI* as previously described.

For DCR1/DCR1S reporter lines, three reporter plasmids were generated in peGFP-1 (Clontech) as diagrammatically shown in Fig. 6A–C. The reporter cassettes were released from the plasmids by digestion with *Mlu*I and *Afl*II and transgenic mice generated by pronuclear injection of FVB/N fertilized eggs at the AECOM Transgenic Core Facility. Founders were genotyped by visualization of GFP expression in the lens using the NightSea Blue Star flashlight (Electron Microscopy Sciences). Embryos were genotyped by PCR using primers against EGFP (F 5′-ACCCTCGTGACCACCCTGACCTAC-3′; R 5′-GACCATGTGATCGCGCTTCTCGTT-3′). Three lines of each mouse were analyzed and demonstrated similar expression patterns (Table 1).

### Immunofluorescence

Embryos were fixed in 4% paraformaldehyde, cryoprotected with 15% sucrose in PBS, and embedded in Optimal Cutting Temperature (OCT) tissue freezing medium (Triangle Biomedical Sciences). Transverse cryostat sections (10 μm) were collected, washed with PBS containing 0.1% triton-X, blocked for 1 h in PBS containing 1% BSA, and then incubated overnight at 4°C with the primary antibody, rabbit anti-GFP (1:1000, Molecular Probes, A-11122), diluted in PBS containing 1% BSA. Sections were washed twice for 10 min in PBS containing 0.1% triton-X and incubated for 45 min with the secondary antibody, goat anti-rabbit Alexa Fluor^®^ 488 (1:500) (Molecular Probes). Slides were washed and mounted with Vectashield containing DAPI (Vector). Images were taken with a Leica AOBS laser scanning confocal microscope.

### Quantitation of EGFP in transgenic mice

RNA was isolated from dissected tissues using miRNesay mini kit (Qiagen) according to manufacturer's instructions. cDNA was generated with Superscript™ III Reverse Transcriptase (Thermo Fisher), and the template was diluted 1:10. Quantitative PCR was performed using SYBR Green (Thermo Fisher) and primers: αA-crystallin (5′-GAGATTCACGGCAAACACAA-3′ and 5′-ACATTGGAAGGCAGACGGTA-3′) and EGFP (5′-ACGACGGCAACTACAAGACC-3′ and 5′-GTCCTCCTTGAAGTCGATGC-3′; 5′-CACATGAAGCAGCACGACTT-3′ and 5′-GGTCTTGTAGTTGCCGTCGT-3′). Primers for αA-crystallin recognize both endogenous and the fusion αA-crystallin-EGFP cDNA. The relative expression level of αA-crystallin was normalized by the EGFP fusion protein average versus endogenous αA-crystallin. Three lines of each mouse were analyzed and EGFP expression values can be seen in Table 1.

### Transgenic copy number analysis

Genomic DNA was isolated by digesting tissue with lysis buffer (100 mM Tris HCL, pH 8.0, 5 mM EDTA, 0.2% SDS, 200 mM NaCl) containing Proteinase K (100 μg/ml) at 55°C. Phenol/chloroform/isoamyl alcohol (Thermo Fisher) extractions were performed and DNA was precipitated with isopropanol. Quantitative PCR was conducted using SYBR Green (Thermo Fisher) to determine the number of copies of the transgene, using the following primers: αA-crystallin (5′-GAGAGGGCCATTCCTGTGT-3′ and 5′-AGGGGACAACCAAGGTGAG-3′); (5′-GGGTGCTGGTCTACTTCCAG-3′ and 5′-AACCACGACATCCGAAAAAG-3′); and CCNI (5′-TCTTCTCCCTCCTCAGACG-3′ and 5′-CCGTTACCACCTCATGATCC-3′); B2M (5′-CCCTGGCTGGCTCTCATT-3′ and 5′-ACTGAAGCGACCGCGACT-3′) for normalization. Three lines of each mouse were analyzed and copy number ranges can be seen in Table 1.

### 3C assay

3C assays were carried out as previously described (Dekker et al., 2002), with some modifications. Approximately 10 mg of tissue was cross-linked in freshly prepared 1% formaldehyde and 10% FBS (vol/vol) in phosphate-buffered saline (PBS) at RT for precisely 10 min with gentle shaking on horizontal shaker. Fixation was stopped by adding 0.125 M final concentration of glycine and incubating for 5 min at RT with gentle shaking on a horizontal shaker. Cells were then pelleted and washed in 1 ml PBS containing 10% (vol/vol) serum at RT. Cells were pelleted once again and lysed in 1 ml lysis buffer [NaCl (100 mM), 50 mM Tris-Cl (pH 8.1), 5 mM EDTA and 1% (w/v) SDS] with freshly added protease inhibitors for 90 min at 4°C with rotation. The nuclei were collected and incubated in 250 μl of restriction buffer 3 (NEB) containing 0.3% SDS at 37°C for 1 h with shaking. The SDS was then sequestered by adding Triton X-100 to 1.8% and incubating at 37°C for another hour with shaking. 100 U of restriction enzyme BstYI were added and incubated overnight at 37°C with shaking. The reaction was stopped by adding SDS to 1.6% and incubating at 65°C for 20 min. The extent of digestion was verified by PCR. 100 μl 10× ligation buffer (NEB), 900 μl dH_2_O and 100 μl 20% Triron X-100 (final vol. 1%) was added and incubated at 37°C for 1 h with shaking. The reaction mixture was then cooled to 16°C and 50 U of T4 DNA ligase (NEB) was added. After 4 h of ligation, the chromatin mixture was incubated with 100 μg/ml proteinase K at 65°C overnight to reverse cross-links. RNA was removed by RNase A (0.5 μg/ml) treatment for 30 min at 37°C and DNA was purified by phenol extraction. Quantitative real-time PCRs were performed, in the presence of SYBR Green (Thermo Fisher), with appropriate primers (sequences available upon request) from purified DNA as well as the control template. The control template was generated by digestion and ligation of αA-Crystallin BAC clone (RP-23-465G4) to generate each ligation product in equal molar amounts. The relative cross-linking frequency between two fragments was calculated by normalizing to the control library. This calculation corrects for differences in cross-linking and ligation efficiencies, PCR amplification efficiency, the amount of the initial template used and the sizes of the PCR products.

### Quantitation of αA-crystallin in mouse tissues

RNA was isolated from P1 mouse lens, liver, forebrain, heart and mouse embryonic stem (mES) cells using miRNesay mini kit (Qiagen) according to manufacturer's instructions. cDNA was generated with Superscript™ III Reverse Transcriptase (Thermo Fisher). Quantitative PCR using SYBR Green (Thermo Fisher) was performed to determine the levels of αA-crystallin and normalized to Gapdh and B2m using the following primers: αA-crystallin (Cryaa: 5′-GAGATTCACGGCAAACACAA-3′ and 5′-ACATTGGAAGGCAGACGGTA-3′); Gapdh (5′-CCAATGTGTCCGTCGTGGATCT-3′; 5′-GTTGAAGTCGCAGGAGACAACC-3′); B2m (5′-CATACGCCTGCAGAGTTAAGC-3′; 5′-GATGCTTGATCACATGTCTCG-3′).

## Supplementary Material

Supplementary information
