# Supplementary material for: Promoter-enhancer looping and shadow enhancers of the mouse αA-crystallin locus
Source: Biol Open. 2018 Nov 7;7(12):bio036897. doi: 10.1242/bio.036897 (PMC6310886; doi:10.1242/bio.036897)
Supplement: Supplementary information [file biolopen-7-036897-s1.pdf]

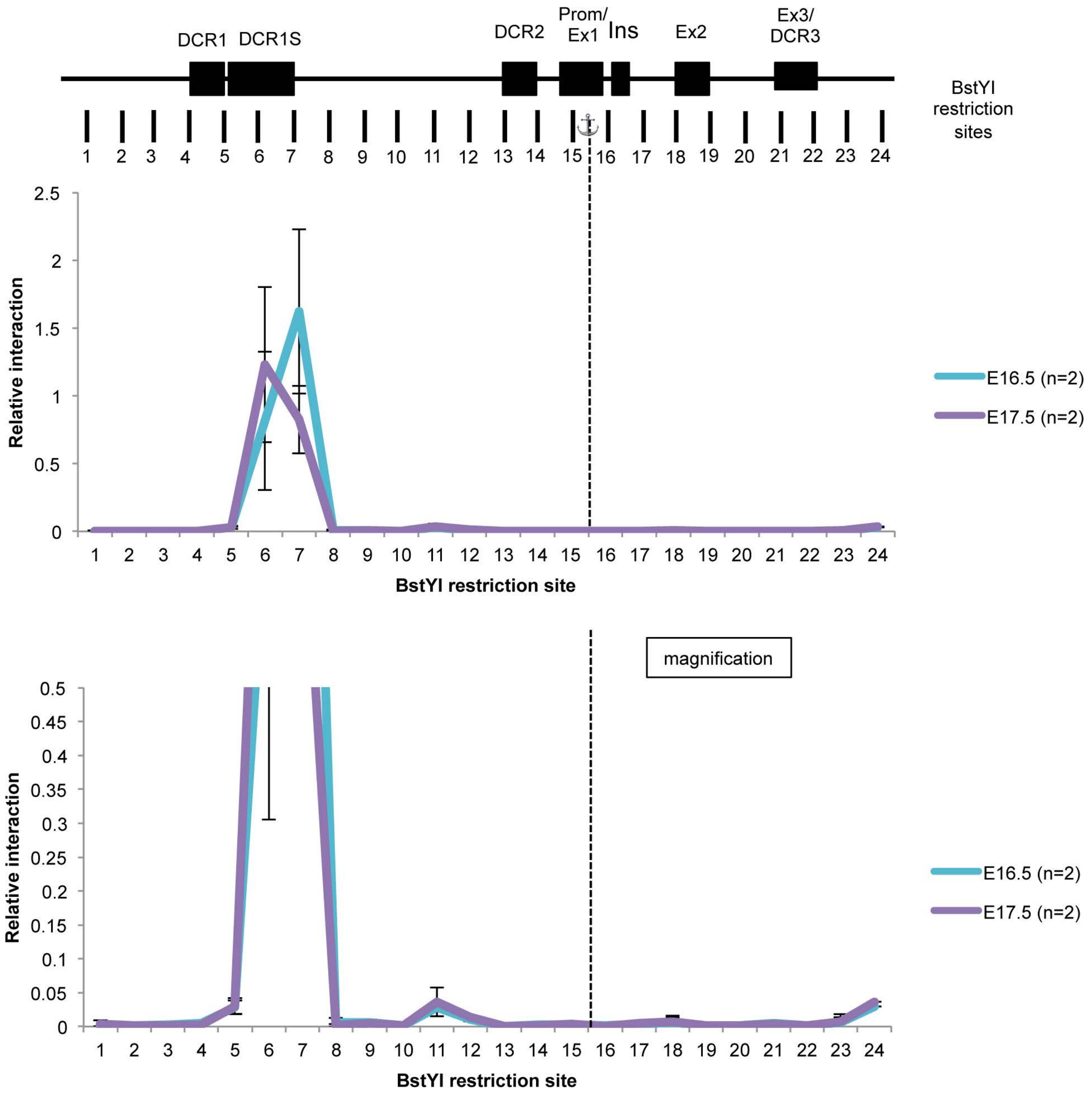
DCR1
DCR1S
DCR2
Prom/
Ex1
Ins
Ex2
Ex3/
DCR3
BstYI
restriction
sites
1 2 3 4 5 6 7 8 9 10 11 12 13 14 15 16 17 18 19 20 21 22 23 24
Relative interaction
2.5
2
1.5
1
0.5
0
E16.5 (n=2)
E17.5 (n=2)
BstYI restriction site
magnification
0.5
0.45
0.4
0.35
0.3
0.25
0.2
0.15
0.1
0.05
0

Figure S1. Chromatin conformation capture (3C) mapping of chromatin interactions in the αA-crystallin locus of E16.5 and E17.5 mouse lens. (A) Genomic organization of the αA-crystallin locus, spanning 16 kb. Dark boxes represent locations of the evolutionarily conserved DCR1, DCR1S, DCR2, the promoter with adjacent exon 1, the rodent specific exon (Ins), exon 2, and exon 3 with adjacent DCR3. Vertical markers represent locations and numbers of BstYI restriction sites analyzed in this study, with the fragments assayed for variable interaction with the promoter fragment (anchor). Graphical representation is not to scale. (B) Relative cross-linking frequency of regions interacting with the αA-crystallin promoter with relative interaction plotted on the y-axis and restriction digest fragment number on the x-axis. (C) Magnification of panel (B) with reduced y-axis scale to identify less frequent interactions. Hashed line shows position of anchor fragment. Each value is derived from two biological samples (n=2) and the standard errors are indicated. Values are normalized to P1=1.0.
